# Steroids as Environmental Compounds Recalcitrant to Degradation: Genetic Mechanisms of Bacterial Biodegradation Pathways

**DOI:** 10.3390/genes10070512

**Published:** 2019-07-06

**Authors:** Elías R. Olivera, José M. Luengo

**Affiliations:** Departamento Biología Molecular (Área Bioquímica y Biología Molecular), Universidad de León, 24007 León, Spain

**Keywords:** sterols, bile acids, steroid hormones, biodegradation, 9,10-*seco* pathway, 4,5-*seco* pathway, 2,3-*seco* pathway

## Abstract

Steroids are perhydro-1,2-cyclopentanophenanthrene derivatives that are almost exclusively synthesised by eukaryotic organisms. Since the start of the Anthropocene, the presence of these molecules, as well as related synthetic compounds (ethinylestradiol, dexamethasone, and others), has increased in different habitats due to farm and municipal effluents and discharge from the pharmaceutical industry. In addition, the highly hydrophobic nature of these molecules, as well as the absence of functional groups, makes them highly resistant to biodegradation. However, some environmental bacteria are able to modify or mineralise these compounds. Although steroid-metabolising bacteria have been isolated since the beginning of the 20th century, the genetics and catabolic pathways used have only been characterised in model organisms in the last few decades. Here, the metabolic alternatives used by different bacteria to metabolise steroids (e.g., cholesterol, bile acids, testosterone, and other steroid hormones), as well as the organisation and conservation of the genes involved, are reviewed.

## 1. Introduction

**Figure 1:**
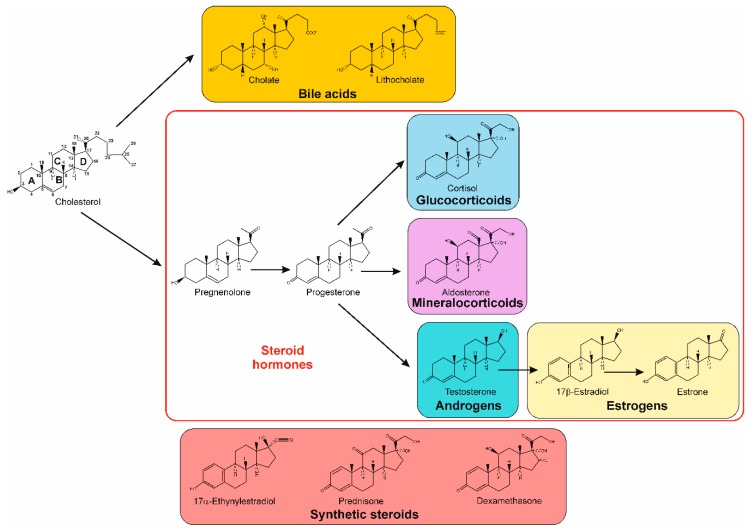
Chemical structure of cholesterol and some of its mammalian derivatives, and selected synthetic steroids.

Steroids are tetracyclic triterpenoid lipids containing a perhydro-1,2-cyclopentanophenanthrene structure, and include sterols, bile acids, steroid hormones, cardenolides, sapogenins, saponins, and vitamin D derivatives. Classically, they have been studied based on their physiological role. In mammals, the steroid hormones, bile acids, and other essential steroids are produced from cholesterol (Figure 1), a molecule that is also an important component of membranes, maintaining their fluidity, and participating in cell differentiation and proliferation. The major constituents of plant sterols are sitosterol, stigmasterol, campesterol, and brassicasterol, while in yeast and filamentous fungi, ergosterol is an important component of cell walls [1,2,3].

It is generally accepted that the biosynthesis of steroids is one of the hallmarks of the evolutionary progression of eukaryotes [4]. However, reports on the biosynthesis of sterols and modified sterols in methanotrophic bacteria (*Methylococcus capsulatus, Methylosphaera hansonii*, and *Methylobacterium organophilum*), *Gemmata obscuriglobus, Eudoraea adriatica*, and in a variety of myxobacteria have been published, though there is limited information about the metabolic machinery involved [5,6,7,8,9,10,11,12,13,14,15].

From an ecological point of view, the release into the environment of vertebrate steroids, such as androgens, estrogens, progestogens, cholesterol, and bile acids, during excreta or decomposition of biomass is constantly occurring [16,17,18,19,20,21,22]. In addition, not only natural steroids are released. Along with antibiotics, synthetic steroids represent a significant sector of the global pharmaceutical market, and they have found their way into environmental niches, as in the case of synthetic hormones (ethynylestradiol and anti-inflammatory drugs like dexamethasone) (Figure 1) [19,20,23,24,25,26,27]. With their widespread presence, they can affect endocrine activity, even at low concentrations, with potential adverse effects for both the environment and human health. As such, there is an increasing need for new approaches in the bioremediation of steroids from the environment.

Although steroid synthesis is mostly restricted to eukaryotic organisms [4], the biodegradation of these compounds seems to be carried out exclusively by bacteria. Even so, steroids are highly recalcitrant to microbial degradation because of the low number of functional groups present in their structure and their extremely low solubility in water. Nevertheless, the persistence of these molecules in the environment has triggered a response by some bacteria, either efficiently detoxifying these hydrophobic molecules or metabolising their carbon and energy-rich scaffold. Thus, the isolation of microorganisms able to degrade or modify steroids has been reported from soil [28,29,30], and from freshwater [31] and marine [32,33,34] environments. This indicates the importance of bacteria in reducing the adverse impact of environmentally released steroids, as well as emphasising their importance in introducing these molecules into the carbon cycle. Moreover, for some pathogenic bacteria, such as *Mycobacterium tuberculosis* and *Rhodococcus equii*, catabolism of cholesterol has been identified as a trait involved in their pathogenicity and persistence in the host [35,36].

In 1913, Söhngen reported that a *Mycobacterium* strain could use phytosterols as a sole carbon and energy source [37]. Thereafter, many bacterial strains have been isolated that are able to degrade steroids, including species initially classified as belonging to genera *Mycobacterium, Gordonia, Tsukamurella, Rhodococcus, Azotobacter, Nocardia, Flavobacterium, Arthrobacter, Bacillus, Brevibacterium, Corynebacterium, Streptomyces, Microbacterium, Serratia, Achromobacter, Protaminobacter*, and *Pseudomonas* [29,38,39,40,41,42,43,44,45,46,47,48,49,50,51,52,53,54]. However, it has not been until the last two decades of the 20^th^ century that studies have been initiated to elucidate the physiological and metabolical determinants, and the mechanisms involved in the aerobic catabolism of different steroid molecules. These studies have included sterols, chiefly cholesterol, testosterone and 17β-estradiol, and bile acids, mainly cholic acid, using selected model organisms from Actinobacteria, comprising different strains of *Mycobacterium, Rhodococcus*, and *Gordonia* [36,55,56,57,58,59] (Table 1), and some selected Gram negative strains, the α-proteobacteria *Comamonas testosteroni* and a few *Pseudomonas* strains [28,29,60,61,62]. In addition, the catabolism of steroids under anoxic conditions has been studied in *Sterolibacterium* (β-proteobacteria) and in *Steroidobacter* (ϒ-proteobacteria) [63,64,65] (Table 1).

## 2. Uptake of Steroids

The catabolism of steroids implies their selective uptake and internalisation inside microbial cells. However, there is limited information about these processes. Regarding steroid uptake, given the physiological and structural surface differences between bacterial groups, its mechanisms in most bacteria have remained elusive. This is especially evident in Gram-negative bacteria, which possess an outer membrane where the lipopolysaccharide leaflet on the outer surface impedes access of steroids to the cytoplasm by passive diffusion [66].

**Figure 2:**
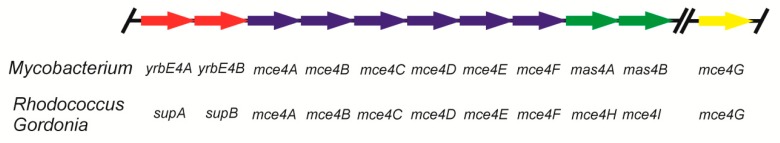
Genetic organisation of the *mce4* cluster involved in sterol uptake in Actinobacteria.

In mycolic acid-containing Actinobacteria, the transport of steroids is carried out by different transport systems, depending on the particular steroid to be assimilated. The sterol uptake into cells of *M. tuberculosis, M. smegmatis, Rhodococcus jostii, R. equi*, and *Gordonia cholesterolivorans* is performed using a set of proteins encoded by the *mce4* locus [35,67,68,69,70,71]. The ten genes contained in the *mce4* locus, referred to in short-hand by the names *yrbE4ABmce4ABCDEFmas4AB* in *Mycobacterium* spp. [71,72,73], and *supABmce4ABCDEFHI* in *Rhodococcus* and *Gordonia* spp. [67,68,69] (Figure 2), codify multicomponent ATP-dependent sterol uptake systems. Thus, these genes are upregulated in *M. tuberculosis, M. smegmatis*, and *R. jostii* during growth on cholesterol [55,73]. The deletion of some or all of the genes of the cluster prevents mutants of *M. smegmatis* [72] and *R. jostii* [67] growing on cholesterol as the sole carbon and energy source. Surprisingly, analogous mutants of *M. tuberculosis* are still able to grow using cholesterol, suggesting such strains have an alternative cholesterol transport system [67].

Further analysis revealed that *yrbE4AB* (*supAB*) encode transmembrane proteins similar to permease components of ABC (ATP-binding cassette) transporters [74]. These putative permease subunits should be anchored into the cytoplasmic membrane to facilitate cholesterol translocation across this cellular barrier. They are associated with Mkl ATPase, encoded by *mceG*, which is not linked with the cluster [35,75] (Figure 2). In *M. tuberculosis*, this protein acts as the ATPase component, not only with Mce4 proteins in the uptake of sterols, but also with Mce1, another transport system that is linked to fatty acid transport rather than to steroid catabolism [35,76]. Even so, both transport systems, Mce1 and Mce4, participate in mycobacterial infection [35,75,76,77,78]. Thus, MceG ATPase hydrolyses ATP, providing energy for the substrate import for both Mce4 in cholesterol uptake and for Mce1 in fatty acid internalisation [35,67]. Mce4ABCDEF are abundant in cell envelope protein fractions, hence their characterisation as cell wall-associated proteins. They probably facilitate cholesterol transport across the mycolic acid layer and/or pseudoperiplasmic space. Signal sequences for protein export via the general secretory pathway can be found in the amino acidic sequences of Mce4ABCDEF. Moreover, Mce4F has a putative transmembrane domain in the N-terminus of the protein, which probably allows for its embedding in the cytoplasmic membrane or cell wall [74,79,80]. In addition, Mas4AB (Mce4HI), both required for cholesterol import [74], have been suggested to be accessory subunits for their respective systems, with a putative function in stabilising or assembling Mce4ABCDEF complexes [76,81].

Other proteins, codified by genes not included in the *mce4* operon, have been connected to cholesterol uptake in *Mycobacterium*. OmamA has been proposed to be a stabiliser of Mce1 and Mce4 complexes, playing a role in cholesterol and fatty acid uptake in this bacterium [81]. In the same way, LucA also participates in the uptake of both cholesterol and fatty acids, having a cell membrane or cell envelope location. LucA interacts with Mas4A, Mas4B, OmamA, and probably MceG, potentially protecting these proteins, and hence the uptake complexes, from proteolytic attack [76].

As previously indicated, the Mce4 system is required for the uptake of sterols in Actinobacteria. However, this system is not used by *R. jostii* when growing on cholic acid as the sole carbon and energy source [82]. The mutation of the gene coding the porin RjpA impairs growth on metabolising cholic acid, however, it had no effect when sterols were the molecules used to support growth, indicating the importance of this porin for cholic acid uptake [82]. Although the system for the entry of this bile acid through plasma membrane has not been identified, a proteomic study of cholic acid growing cells suggested there might be two systems, CamM, a major facilitator superfamily transporter, and CamABCD, an ATP-binding cassette (ABC) transporter [83]. However, a parallel study demonstrated that both systems act in the reassimilation of transiently accumulated cholic acid intermediates, previously secreted to the culture broth by bacteria during bile acid catabolism [84]. The accumulation of metabolic intermediates in culture broths by steroid-degrading bacteria has been documented [85]; *Pseudomonas stutzeri* strain Chol1 metabolising bile acids extracellularly accumulates intermediates, probably to regulate their intracellular levels or due to overflowing of the downstream metabolic pathway [85]. Later, during growth, these metabolites disappear from the culture broth, which implies: (i) the existence in these bacteria of an exclusion system, and (ii) a catabolic route involved in the reassimilation of these compounds [85].

In the anaerobic cholesterol-degrading *Sterolibacterium denitrificans* DSMZ 13999, it has been suggested that assimilation of cholesterol could proceed via direct adhesion, followed by outer membrane transport mediated by a FadL-like transport system. Once in the periplasm, initial reactions in cholesterol catabolism occur. Notably, the Fad-like system exhibited high substrate specificity for C27 sterols, but not for C19 androgens [86].

## 3. The Aerobic 9,10-*seco* Pathway

The most complete biochemical and genetic information about steroid catabolic pathways is based on the investigation of a few Actinobacteria and Proteobacteria species, which can mineralise steroids under aerobic conditions. Thus, testosterone and bile acid degradation have been studied in *C. testosteroni* strain TA441 and *C. thiooxidans* CNB-1 [60], *P. stutzeri* strain Chol1 [85,87], and *Pseudomonas putida* strain DOC21 [29,62]. Cholesterol degradation has been studied in *R. jostii* strain RHA1 [55], *R. equi* strain 103S [36], *Rhodococcus rhodocrous* strain DSM 43269 [88], *Rhodococcus ruber* strain Chol-4 [54], *M. tuberculosis* strain H37Rv [89,90], *M. smegmatis* strain mc^2^ 155 [56,91], *G. cholesterolivorans* strain Chol-3 [53,69], and *Gordonia neofelifaecis* strain NRRL B-59395 [59,92]. Bile acid degradation has also been studied in *R. jostii* RHA1 [57,84]. In all these bacteria, the degradation of the chemical nucleus of the different steroids follows similar steps, using the 9,10-*seco* pathway. Results from the laboratory of Mohn [34,93] suggest that only Actinobacteria degrade sterols with the 9,10-*seco* pathway, while Proteobacteria degrade bile acids and other less structurally complex steroids using this pathway.

### 3.1. Oxidation of 3-hydroxyl-substituent from Sterols and Bile Acids

The degradation of steroid compounds by the 9,10-*seco* pathway is initiated by oxidative attack of 3-oxo steroid. Therefore, steroids, such as sterols or bile acids, carrying a 3-hydroxyl-substituent, need to be oxidised to the corresponding ketone as a preliminary step. However, in *R. jostii* strain RHA1 it has been reported that the catabolism of sterols starts through the ω-oxidation of the C_17_ side chain to the corresponding carboxylic acid, which is later catabolized through the classic pathway (see below). However, it is not clear if the formation of the carboxylic acid occurs before or simultaneously with the initial oxidation of the sterane rings [94].

**Figure 3:**
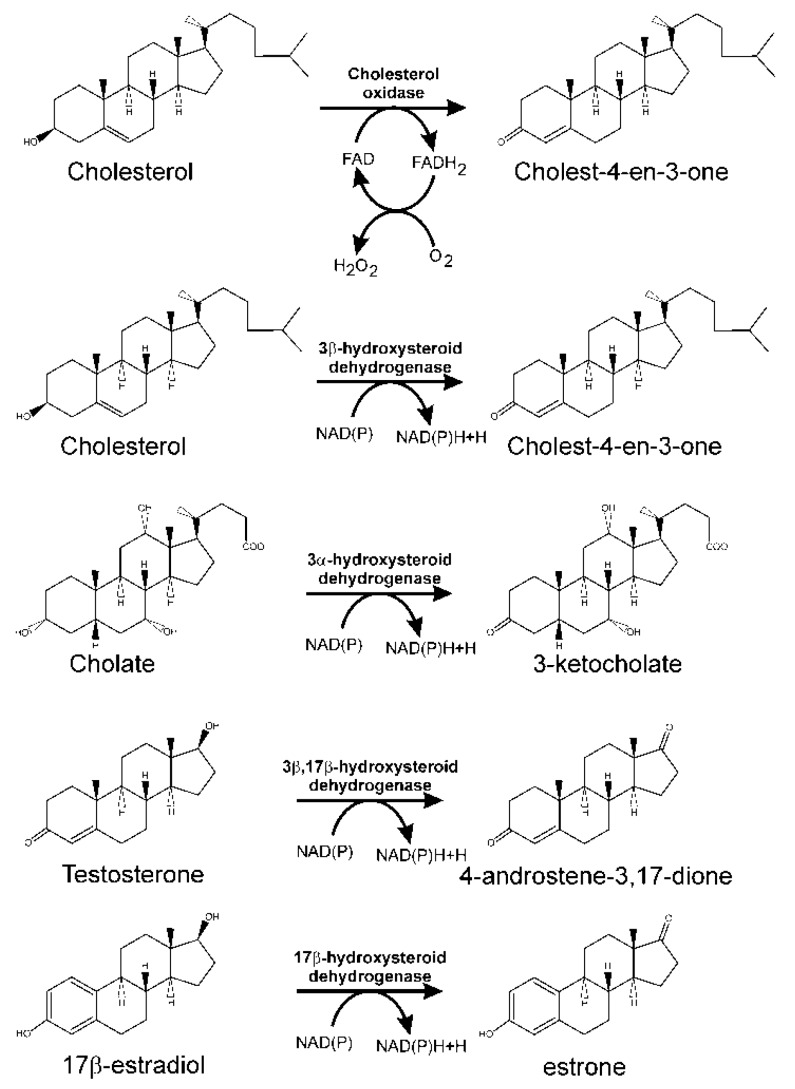
Initial reactions catalysed by cholesterol oxidase and different hydroxysteroid dehydrogenases in steroid degradation through the 9,10-*seco* pathway.

For sterols, the initial reaction involves the oxidation of the 3β-hydroxy group, concomitantly with isomerisation of the C5–C6 double bond present in these molecules, to yield 3-keto-4-ene structures. Thus, considering cholesterol as a sterol model, the first reaction in its catabolism results in its transformation into cholest-4-en-3-one. This double step, oxidation of the hydroxyl group and isomerisation of the double bond, is catalysed either by a cholesterol oxidase requiring molecular oxygen or by NAD(P)-dependent 3-β-hydroxy-Δ^(5)^-steroid dehydrogenase (Figure 3) [95].

Most of the organisms that possess a cholesterol oxidase produce it as an extracellular form, either released into the culture broth or linked to the cell surface. Cholesterol oxidases are monomeric enzymes containing FAD, and belong to two different classes. Class I includes enzymes, belonging to the glucose/methanol/choline oxidoreductase family, which fix FAD into their active site cavity through non-covalent bonds [96]. However, Class II enzymes belong to the vanillyl-alcohol oxidase family, covalently binding the FAD cofactor [97].

A NAD(P)-dependent cholesterol dehydrogenase has been isolated from cells of *Nocardia* sp. Ch2-1 [98]. In *M. tuberculosis*, the formation of cholest-4-en-3-one is carried out by a 3-β-hydroxysteroid dehydrogenase (HsdD, Rv1106c) [99]. Although a gene for cholesterol oxidase (ChoD, Rv3409c) has also been found in this bacterium, its activity has not been confirmed in vitro, and its homology to many described cholesterol oxidases is rather limited. Moreover, its disruption does not result in a marked change in the ability of the mutant strain to grow using cholesterol [100].

In *M. smegmatis*, a constitutive cholesterol oxidase (Msmeg1604) similar to ChoD from *M. tuberculosis*, and two cholesterol inducible enzymes, Msmeg5228 (a cholesterol oxidase) and Msmeg5233 (a cholesterol dehydrogenase/isomerase), have been identified. Msmeg1604 does not seem to play a critical role in the mineralisation of cholesterol, since a specific mutation in this protein does not affected the production of cholest-4-en-3-one. However, an Msmeg5228 defective mutant shows a drastic reduction in the formation of this intermediate [56]. In addition, double mutants affecting HsdD and ChoD in *M. tuberculosis*, and Msmeg5228 and Msmeg5233 in *M. smegmatis*, are still able to grow on cholesterol, suggesting there are other dehydrogenase/isomerases that could replace them in the first reaction of the cholesterol degradation pathway [56,101].

In *R. ruber*, Chol-4, a constitutive extracellular cholesterol oxidase has been characterised [58]. However, although the deletion of this gene delayed bacterial growth when cholesterol was the sole carbon source, it did not completely prevent it, suggesting the existence of genes with overlapping activities in this bacterium [58].

Bile acids also have a hydroxyl group in the C_3_ position that should be oxidised to a 3-oxo group prior to oxidation of the steroid nucleus. However, this hydroxyl group is in an α-configuration, and its oxidation in those strains able to use bile acids as a carbon and energy source is performed by 3α-hydroxysteroid dehydrogenase, which is a NAD(P)-dependent enzyme belonging to the short chain dehydrogenase/reductase superfamily [102]. The first description of this kind of enzymes corresponds to a 3α-hydroxysteroid dehydrogenase/carbonyl reductase in *C. testosteroni* [103]. It was found to be functional as an oxidoreductase towards a variety of 3α-steroid substrates [104,105]. The enzyme also catalyses the reduction of non-steroidal aldehydes and ketones, and consequently, has been named 3α-hydroxysteroid dehydrogenase/carbonyl reductase [105]. Its gene has been cloned from *C. testosteroni* ATCC 11996 [106], and it is also involved in steroid catabolism in *C. testosteroni* strain TA441 [107].

**Figure 4:**
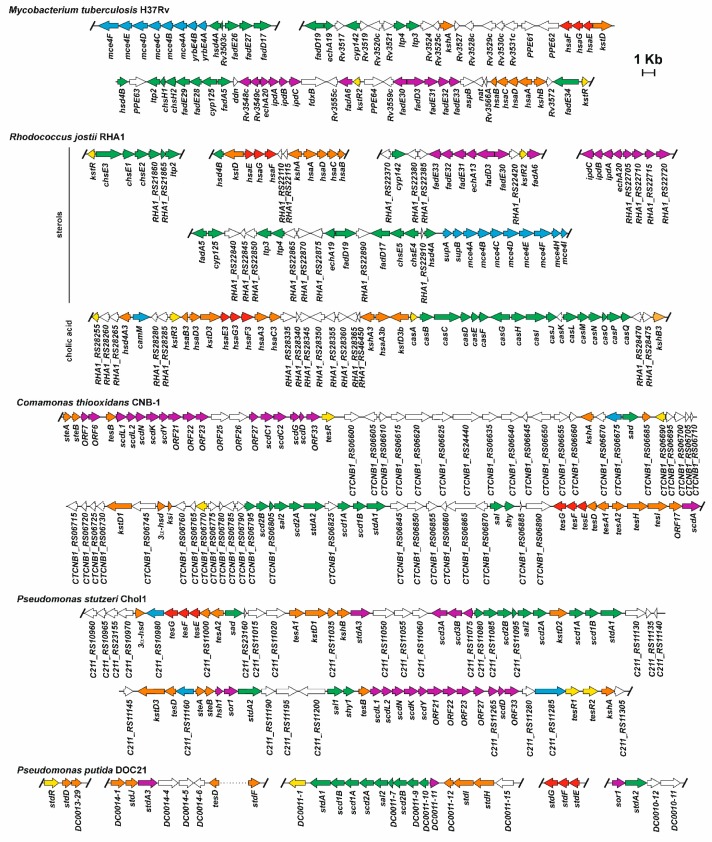
Genetic organisation of the genes encoding the 9,10-*seco* pathway involved in cholesterol (*Mycobacterium tuberculosis* H37Rv, *Rhodococcus jostii* RHA1), or in cholic acid and testosterone (*R. jostii* RHA1, *Comamonas thiooxidans* CNB-1 (formerly, *Comamonas testosteroni* CNB-2), *Pseudomonas stutzeri* Chol1 (formerly *Pseudomonas* sp. Chol1), and *Pseudomonas putida* DOC21 catabolism. Genes coding enzymes involved in cholesterol or bile acids side chain degradation are shown in green; genes participating in ring A/B degradation are in orange; in purple are shown genes coding for ring C/D degradation; blue color indicates genes coding transport systems.

NAD-dependent 3α-hydroxysteroid dehydrogenase activity has been detected in *P. stutzeri* Chol1 cell-free extracts when this bacterium was cultured in a medium containing cholic acid as the carbon source [85]. Moreover, the encoding gene has been annotated in the draft genome of this bacterium within the 79-Kb gene cluster containing the ORFs required for steroid assimilation (Figure 4) [108].

In *P. putida* DOC21, a Tn5 insertion in a gene encoding a 3α-hydroxysteroid dehydrogenase revealed that this mutant was unable to further metabolise any of the tested bile acids, cholic, lithocholic, chenodeoxycholic, ursodeoxycholic, and deoxycholic acid [62].

### 3.2. Side Chain Degradation of Sterols and Bile Acids

**Figure 5:**
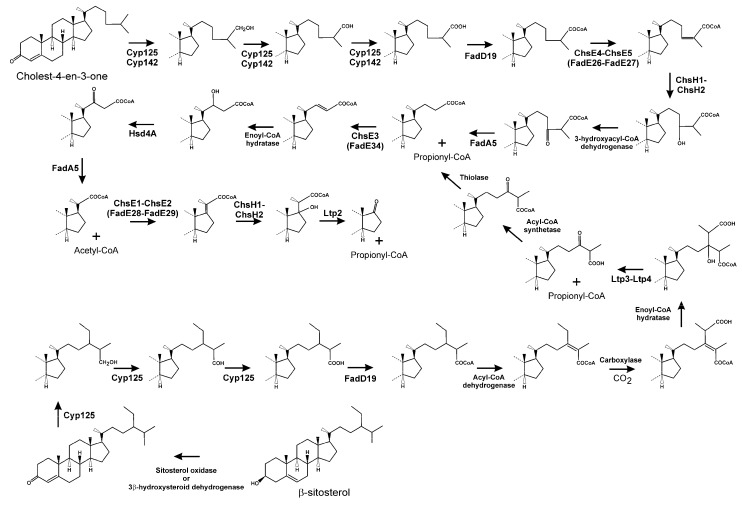
Catabolism of the cholesterol side chain and C_24_-branched chain of β-sitosterol in Actinobacteria.

After this initial step, the catabolism of steroids proceeds through two sub-pathways, which involve C_17_ side chain cleavage and/or steroid nucleus oxidation. Degradation of the alkane side chain of cholesterol has been proposed to proceed through a β-oxidation-like process analogous to that of the catabolism of fatty acyl-CoA in human mitochondria and peroxisomes (Figure 5) [109,110,111]. Thus, cholest-4-en-3-one is used as substrate by Cyp125 P450 cytochrome, catalysing the oxidation of the C_26_ or C_27_ terminal methyl group [94,112,113,114], followed by further oxidation to provide the initial carboxylate. Although in *M. tuberculosis* strain CDC1551 this protein is absolutely required for bacterial growth on cholesterol as the sole carbon source [115], in *M. tuberculosis* strain H37Rv, loss of this activity is compensated for by another P450 cytochrome, Cyp142 [116]. However, substrate specificities of the two P450 cytochromes are different, with Cyp125 being specific for cholesterol, and although able to oxidise cholesteryl sulfate at a low rate, it is unable to oxidise cholesteryl propionate. By contrast, Cyp142 can efficiently metabolise cholesteryl-sulfate as well as cholesteryl-propionate. This suggests that Cyp142 enzymes may play an important role during *M. tuberculosis* infection, by providing access to additional reservoirs of esterified intracellular cholesterol that would not otherwise be available to the pathogen [117].

The carboxyl-functionalised side chain is then converted to its CoA thioester by FadD19, producing 3-oxocholest-4-en-26-oyl-CoA (Figure 5) [118,119]. Later, an α_2_β_2_ heterotetrameric acyl-CoA dehydrogenase, ChsE4-ChsE5, also named FadE26-FadE27, catalyses the α,β-unsaturation of this acyl-CoA thioester, to form 3-oxocholest-4,24-dien-26-oyl-CoA (Figure 5) [90,120,121]. Notably, those human and bacterial acyl-CoA dehydrogenases involved in β-oxidation form α4 homotetramers or α2 homodimers [122], in contrast to the unusual quaternary structure of acyl-CoA dehydrogenases acting in cholesterol side chain catabolism. 3-Oxocholest-4,24-dien-26-oyl-CoA is the substrate of a MaoC-like enoyl-CoA hydratase, ChsH1-ChsH2, being converted into 24-hydroxy-3-oxocholest-4-en-26-oyl-CoA (Figure 5) [123]. Transformation of this compound to 3,24-dioxocholest-4-en-26-oyl-CoA should be carried out by a β-hydroxyacyl-CoA dehydrogenase. Although there is no experimental evidence, it has been proposed that Hsd4A in *M. tuberculosis* could be the enzyme involved in this process [124,125]. Next, FadA5, a thiolase, catalyses the cleavage of this last CoA-esterified oxo-derivative, forming 3-oxochol-4-en-24-oyl-CoA and propionyl-CoA (Figure 5) [126,127]. FadA5 has been categorised as a member of the trifunctional enzyme-like thiolases, type-1 class, which contains a predicted binding site for a bulky fatty acid tail [128,129]. At this point, a second β-oxidation cycle starts, with the introduction of a *trans* double-bond in 3-oxochol-4-en-24-oyl-CoA by ChsE3 (FadE34), followed by the hydration of the double bond by an as yet unknown enoyl-CoA hydratase, and then a dehydrogenation performed by HsdA4, generating 3,22-dioxochol-4-en-24-oyl-CoA [130]. Finally, FadA5 acts again, releasing acetyl-CoA and 3-oxo-pregne-20-carboxyl-CoA (Figure 5) [124,126,127].

FadA5 has been proposed as the thiolase involved in finishing the first two β-oxidation-like rounds. However, although the gene encoding this activity has been shown to be upregulated in the presence of cholesterol in *M. tuberculosis* strain H37Rv, the *fadA5* homologue in *R. jostii* strain RHA1 was not similarly upregulated [55]. Conversely, when *fadA5* was inactivated in a *R. rhodochrous* DSM 43269 derivative, the resulting mutant was not impaired in cholesterol or β-sitosterol side chain degradation, indicating that FadA5 is not essential for the degradation of the sterol side chain in this strain [126,131].

The elimination of the side chain ends by a mechanism resembling a new β-oxidation cycle (Figure 5), starting with dehydrogenation of 3-oxo-4-pregnene-20-carboxyl-CoA to 3-oxo-4,17-pregnadiene-20-carboxyl-CoA in a process catalysed by another heterotetrameric α_2_β_2_ acyl-CoA dehydrogenase complex, ChsE1-ChsE2 (FadE28-FadE29; Figure 5) [90,120,121]. This last compound is then the substrate of an enoyl-CoA hydratase (ChsH1-ChsH2) [90], and the hydrated molecule undergoes an aldol-lyase cleavage reaction catalysed by Lpt2, producing androst-4-en-3,17-dione (AD) and releasing another propionyl-CoA molecule (Figure 5) [90,109,110,132]. Ltp2, at the protein sequence level, is more related to thiolases and acetoacetyl-CoA synthases than to aldolases from other metabolic pathways. It has been shown that Lpt2 interacts with ChsH2, forming a complex ChsH1-ChsH2-Ltp2, catalysing the last two steps of side chain removal from cholesterol [132]. In summary, the complete metabolism of the cholesterol side chain results in a 17-keto steroid intermediate, AD, as well as one acetyl-CoA and two propionyl-CoA molecules.

Removal of the C_17_ side chains of β-sitosterol and campesterol are processes less characterised than cholesterol side chain degradation. Both β-sitosterol and campesterol contain branched side chains that impede the first β-oxidation round of the side chain; therefore, these need to be eliminated to allow full degradation of the side chain. Initially, the C_24_-branched side chain is oxidised at position C_26_ by Cyp125, followed by CoA activation by FadD19 (Figure 5) [118]. Next, the bond between C_24_/C_25_ is desaturated. Removal of the C_24_-branches then starts by carboxylation of the C_28_ carbon, followed by a hydration reaction of the double bond, and finally the release of acetyl-CoA, from campesterol, or propionyl-CoA, from β-sitosterol, via cleavage of the C_24_–C_25_ bond by a heteromeric aldol-lyase (Ltp3-Ltp4) (Figure 5) [131]. It is evident that after aldolytic cleavage of the branch, thioesterification of the resulting carboxylate should be mandatory for degradation of the side chain (Figure 5). At the end of side chain degradation from β-sitosterol and campesterol, AD is formed.

**Figure 6:**
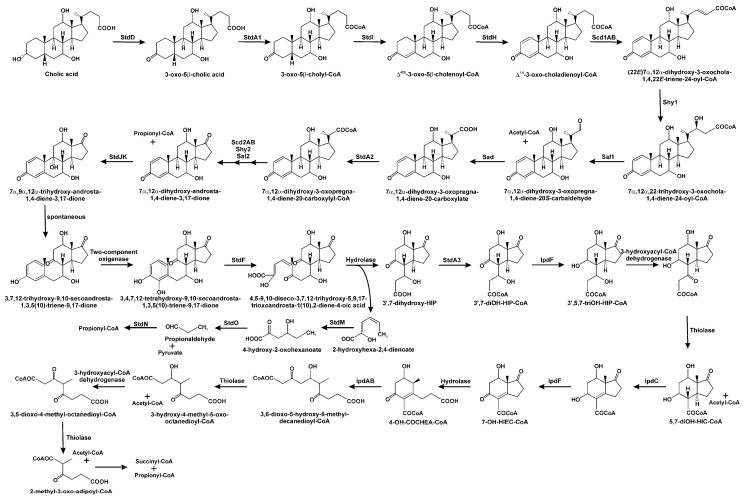
Cholic acid metabolism in *Pseudomonas putida* DOC21 and *P. stutzeri* Chol1 through the 9,10-*seco* pathway. Preliminary evidence suggests that oxidation of the A-ring occurs simultaneously with C_17_ side chain degradation. Hydroxyl groups at C_7_ and C_12_ are maintained during degradation of the molecule, the affecting 3aα-H-4α(3′-propanoate)7a-β-methylhexahydro-1,5- indanedione-hydroxylated derivatives metabolism. 3′,7-diOH-HIP, 3aα-*H*-4α(3′(*R*)-hydroxy-3′propanoate)-7-hydroxy-7aβ-methylhexahydro-1,5-indanedione; 3′,7-diOH-HIP-CoA, 3aα-*H*-4α(3′(*R*)-hydroxy-3′propanoyl-CoA)-7-hydroxy-7aβ-methylhexahydro- 1,5-indanedione; 3′,5,7-triOH-HIP-CoA, 3aα-*H*-(3′(*R*)-hydroxy-3′propanoyl-CoA)-5,7-dihydroxy-7aβ-methylhexahydro-1-indanone; 4OH-COCHEA-CoA, 2-(2-carboxyethyl)-4-hydroxy-3-methyl-6-oxocyclohex-1-ene-1-carboxyl-CoA; 7-OH-HIEC-CoA, (7a*S*)-7a-methyl-7-hydroxy-1,5-dioxo-2,3,5,6,7,7a-hexahydro-1*H*-indene-4-carboxyl-CoA; 5,7-diOH-HIC-CoA, 3aα-*H*-4α(3′-carboxyl-CoA)-5,7-dihydroxy-7aβ-methylhexahydro-1-indenone.

Catabolism of the side chain of bile acids is a process in which not all the enzymatic steps are fully characterised. After oxidation of their hydroxy-group at C_3_ to an oxo-group in these carboxylated molecules, the degradation of the lateral chain begins. Firstly, the carboxy group from the side chain in C_17_ is activated to a coenzyme A thioester through a reaction catalysed by a bile acid-CoA ligase (StdA1 in *P. putida* DOC21 and CasG in *R. jostii*; Figure 6) [56,62,118]. Once activated, a β-oxidation-like process occurs, although it is different to the two initial rounds in the cholesterol side chain degradation. In the first place, a dehydrogenation of C_2_/C_3_ of the acyl-CoA side chain occurs. Based on bioinformatics analysis of clusters coding for bile acid catabolism in *P. stutzeri* Chol1, the participation of a heteromeric acyl-CoA dehydrogenase, Scd1AB, has been proposed [108]. In a second step, the hydration of the α,β-double bond takes place. The introduction of a hydroxy group at the third carbon of the lateral chain in *P. stutzeri* Chol1 is catalysed by the enoyl-CoA hydratase Shy1 (Figure 6) [87]. Until this step, the process is identical to a canonical β-oxidation. However, the next enzymatic step is not the expected oxidation of the hydroxy function to a keto group, followed by a thiolytic cleavage mediated by the introduction of a CoA molecule; instead, it is an aldolic cleavage of the C–C bond, catalysed by Sal1, generating an aldehyde and releasing an acetyl-CoA molecule (Figure 6) [87,133]. Thus, cleavage of these two carbons in unbranched side chains of bile acids proceeds through a retroaldol reaction, as a retro-Claisen reaction, instead of the typical reaction catalysed by thiolases. The resulting aldehyde is then oxidised to the corresponding carboxylic acid by a specific aldehyde dehydrogenase (Sad in *P. stutzeri* Chol1; Figure 6) [87], and later, a second acyl-CoA synthetase (StdA2 in *P. putida* DOC21 and CasI in *R. jostii*) activates it to a CoA derivative [56,62,119]. The subsequent degradation of the remaining side chain is believed to occur through a similar mechanism, where an acyl-CoA dehydrogenase, putatively Scd2AB, introduces an α,β-desaturation in the CoA-activated C_3_ side chain (Figure 6) [85,108]. Hydration of the double bond takes place, resulting in the formation of a hydroxyl group at C_17_, and then an aldolytic cleavage of the molecule occurs, yielding a molecule of propionyl-CoA and a steroid derivative with a keto function at C_17_ (Figure 6). The enzymes catalysing these two steps have not been fully characterised yet [108].

### 3.3. Testosterone Catabolism Convergence

For the catabolic convergence of testosterone into this pathway, the 17β-hydroxy substituent needs to be oxidised to a 17-oxo-derivative. The oxidation of this substituent to a keto group produces androst-4-en-3,17-dione [134]. Currently, the best characterised bacterial enzyme performing this reaction is 3β,17β-hydroxysteroid dehydrogenase (3,17β-HSD) from *C. testosteroni*, encoded by the gene *βhsd* (Figure 3). This NAD(H)-dependent enzyme belongs to the short-chain dehydrogenases/reductases superfamily. It was first purified from strain ATCC11996, and was initially defined as 3β-HSD; however, the cloned enzyme was revealed to act on both the 3β-hydroxyl and 17β-hydroxyl groups of androgens, estrogens, and iso-bile acids [134,135,136]. Horinouchi et al. [107] suggested that the main role of 3,17β-HSD in *C. testosteroni* TA441 cells is 3β-dehydrogenation, and there is at least one more dehydrogenase acting on the 17β-hydroxyl group, since a knock out mutation in the coding gene did not prevent its growth on testosterone. Thus, *C. testosteroni* may have more than one enzyme with 17β-dehydrogenating/hydrogenating activities, to deal with intermediate compounds having considerable structural differences [107]. However, the degradation of testosterone by the 3,17β-HSD mutant is highly affected. Moreover, 3,17β-HSD gene expression is highly induced by testosterone, but not by estradiol and cholesterol, suggesting that this enzyme is a key component in the degradation of testosterone [137].

In *P. putida* DOC21, a microorganism isolated from soil based on its capacity to degrade bile acids and testosterone, a gene encoding 17β-hydroxysteroid dehydrogenase has been identified (unpublished results). This enzyme only has the ability to oxidise the 17β-hydroxy-group of testosterone, because, although this bacterium efficiently metabolises testosterone and androstanolone, it is unable to metabolise closely related compounds with a 3β-hydroxy group (i.e., *trans*-androsterone) [29]. However, current studies are being performed to more completely characterise this enzyme. Other bacterial 17β-hydroxysteroid dehydrogenases have been identified; however, they have been linked to estrogen degradation (Figure 3) through the 4,5-*seco* pathway (see below).

### 3.4. Oxidation of A/B Rings

**Figure 7:**
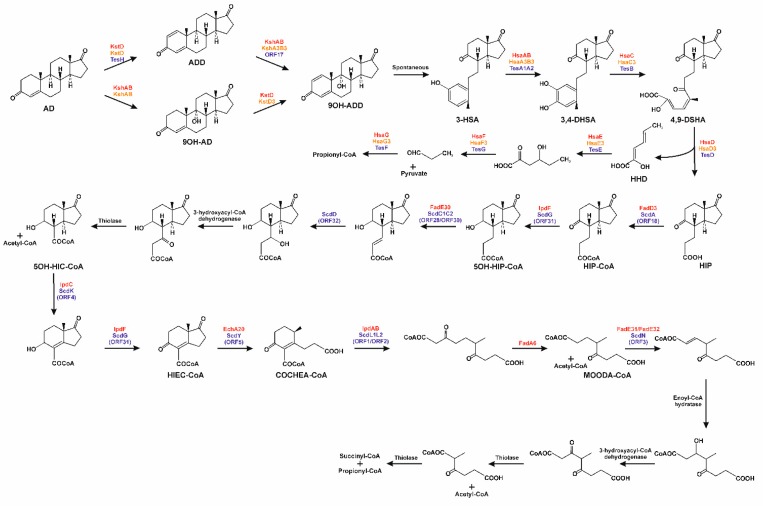
Proposed 9,10-*seco* pathway from androst-4-en-3,17-dione to central metabolites, pyruvate, acetyl-CoA, propionyl-CoA, and succinyl-CoA. AD, androst-4-en-3,17-dione; ADD, androst-1,4-dien-3,17-dione; 9OH-AD, 9-hydroxy-androst-4-en-3,17-dione; 9OH-ADD, 9-hydroxy-androst-1,4-dien-3,17-dione; 3-HAS, 3-hydroxy-9,10-secoandrosta-1,3,5(10)-triene-9,17-dione; 3,4-DHSA, 3,4-dihydroxy-9,10- secoandrosta-1,3,5(10)-triene-9,17-dione; 4,9-DSHA, 4,5,9,10-diseco-3-hydroxy-5-9-17- trioxoandrosta-1(10),2-diene-4-oic acid; HHD, 2-hydroxy-2,4-hexadienoic acid; HIP, 3aα-H-4α(3′-propanoate)7a-β-methylhexahydro-1,5-indanedione; 5OH-HIP-CoA, 3aα-H-4α(3′-propanoyl-CoA)-5-hydroxy-7a-β-methylhexahydro-1-indanone; 5OH-HIC-CoA, 3aα-H-4α(3′-carboxyl-CoA)-5-hydroxy-7a-β-methylhexahydro-1-indanone; HIEC-CoA, (7a*S*)-7a-methyl-1,5-dioxo-2,3,5,6,7,7a-hexahydro-1*H*-indene-4-carboxyl-CoA; COCHEA-CoA, 2-(2-carboxyethyl)-3-methyl-6-oxocyclohex-1-ene-1-carboxyl-CoA; MOODA-CoA, 4-methyl-5-oxo- octanedioyl-CoA. Actinobacterial enzymes from cholesterol metabolism are indicated in red, actinobacterial enzymes involved in cholic acid catabolism are indicated in orange, and those from catabolism of testosterone and cholic acid from *Comamonas* spp. are written in blue.

Because of the initial processing of the steroid nucleus and elimination of the C_17_ side-chain, a common intermediate, AD, arises from sterols and testosterone. Starting from this compound, a common pathway is used, under aerobic conditions, for the complete assimilation of carbon atoms with the concomitant production of metabolic energy (Figure 7). This starts with the transformation of AD into androst-1,4-dien-3,17-dione (ADD), by the desaturation of the bond between C_1_ and C_2_, with trans axial removal of the hydrogen atoms C_1_ (α) and C_2_ (β), in a reaction catalysed by a 3-ketosteroid-Δ^1(2)^-dehydrogenase [138,139]: TesH in *C. testosteroni* [140], StdH in *P. putida* DOC21 [141], KstD in *M. tuberculosis* and *Rhodococcus erythropolis* [142,143,144,145], and KsdD in *M. smegmatis* [146].

For the degradation of bile acids in *P. putida* DOC21, concomitant with the elimination of the C_17_ side chain, oxidation of the ring A starts with desaturation at C_4_ (catalysed by a 3-ketosteroid-Δ^4(5α)^-dehydrogenase, StdI) and at C_1_ (catalysed by StdH; Figure 6) [62,141]. After the elimination of the acyl-side chain and the introduction of two double bonds in the A ring of the cholic acid molecule, 7α,12α-dihydroxy-androsta-1,4-diene-3,17-dione is obtained (Figure 6). Notably, these hydroxyl substituents are maintained during the catabolic process (see below). However, the degradation of lithocholic acid (lacking the hydroxy groups at C_7_ and C_12_) results in ADD, which is a catabolite of convergence when testosterone and AD degradation occurs. Thus, ADD, as well as the hydroxylated derivatives generated from other bile acids, seems to represent a convergence point for the degradative pathway of steroids in *Pseudomonas* species.

Moreover, a 3-ketosteroid-Δ^4(5a)^-dehydrogenase, TesI, has been identified in *C. testosteroni* strains ATCC17410 and TA441 (Figure 4) [107,147]. This enzyme is needed by this bacterium to efficiently catabolise androsterone, androstanolone, androstenedione, and bile acids. Along with its gene sequence, this enzyme has also been characterised in *R. jostii* RHA1 [148]. It is interesting to note that *stdI* and *stdH* from *P. putida* DOC21, as well as *tesH* and *tesI* from *C. testosteroni* TA441, are adjacent to each other in their respective genomes, being part of the steroid-degrading gene clusters (Figure 4) [60,141].

Once ADD, or the hydroxylated derivatives originating from catabolism of different bile acids catabolism, has been synthesised, a monooxygenase/reductase complex (3-ketosteroid 9α-hydroxylase) introduces an α-hydroxy group at C_9_ with KshAB (Figure 7) in *R. erythropolis* [149,150] and in *M. tuberculosis* [151,152]. The resulting 9-hydroxy-androst-1,4-dien-3,17-dione is an unstable molecule that undergoes abiotic cleavage of the B-ring and aromatisation of the A-ring to form 3-hydroxy-9,10-secoandrosta-1,3,5(10)-triene-9,17-dione (Figure 7).

While the formation of 9α-hydroxy-4-androstene-3,17-dione, and later desaturation in the A ring has been described, it is worth mentioning that the order of desaturation and hydroxylation reactions in Actinobacteria is unclear [149,150,151]. Moreover, the introduction of desaturation between C_1_ and C_2_ has been proposed to occur at different stages of side chain degradation in some Actinobacteria, i.e., in *R. ruber* Chol4, *R. erythropolis* SQ1, and different strains of *Nocardia, Arthrobacter*, and *Mycobacterium* [153,154]. In fact, different 3-ketosteroid-Δ^1(2)^-dehydrogenase paralogs showing differences in their substrate specificities have been characterised in some of these strains. For example, in *R. ruber* Chol-4, three different KstD isoenzymes (KstD1, KstD2, and KstD3) differing in their respective substrate profiles have been described, with KstD2 being the isoenzyme mainly involved in AD degradation in this strain [155]. In *Mycobacterium neoaurum* ATCC 25795, three different paralogs of this dehydrogenase have also been identified. In this strain, KstD1 showed a higher affinity for 9α-hydroxy-4-androstene-3,17-dione, while KstD3 preferred AD [156].

In Actinobacteria, it is not surprising to find different paralog genes coding KshA subunits, ranging from one to six. In *M. tuberculosis* H37Rv, only one *kshA* homolog has been found, in *Mycobacterium* spp. VKM Ac-1815D and 1816D, two different versions have been reported, and in *Mycobacterium* VKM Ac-1817, five different paralogs have been described [157,158]. In *R. rhodochrous* DSM43269 and *R. erythropolis* SQ1 chromosomes, five different paralogs of *kshA* have been identified [157,159], and the coded proteins showed different substrate specificities. Thus, KshA5 from *R. rhodochrous* appears to have the broadest substrate range, but without a clear substrate preference. By contrast, KshA1 seems to be specifically involved in cholic acid catabolism [159]. The reductase component of 3-ketosteroid 9α-hydroxylase in Actinobacteria, KshB, is generally present as a single copy gene, with some exceptions for those bacteria able to degrade sterols and bile acids, suggesting that each copy of this gene could be involved in the specific degradation of a particular compound.

Conversely, steroid-degrading Proteobacteria seem to only have single copies of 3-ketosteroid-Δ^1(2)^-dehydrogenase and both subunits of 3-ketosteroid 9α-hydroxylase, suggesting a possible reason for their rare ability to catabolise a wide range of steroids. Although it has been proposed that the multiplicity of genes encoding 3-ketosteroid-Δ^1(2)^-dehydrogenase and the oxygenase subunits of 3-ketosteroid 9α-hydroxylase in some Actinobacteria could represent an environmental advantage, their role could also be in the maintaining metabolic flux throughout the pathway, avoiding the accumulation of intermediates, and so facilitating a dynamic and fine-tuned steroid catabolism.

Catabolism continues through the hydroxylation of the aromatised A-ring in 3-hydroxy-9,10-secoandrosta-1,3,5(10)-triene-9,17-dione by a two-component oxygenase, TesA1A2 in *C. testosteroni* [160], and HsaAB in *M. tuberculosis* and *R. jostii* RHA1 [161], leading to the formation of a catecholic derivative, 3,4-dihydroxy-9,10-secoandrosta-1,3,5(10)-triene-9,17-dione (Figure 7). In this type of monooxygenase, the reductase utilises NADH to reduce a flavin, which is then transferred to the oxygenase. The dihydroxylated ring is then opened by *meta*-cleavage by an extradiol dioxygenase (TesB in *C. testosteroni* [162], and HsaC in *M. tuberculosis* [163] and *R. jostii* RHA1 [55]) that introduces two oxygen atoms. In *P. putida* DOC21 the coding gene for this meta-cleavage dioxygenase, *stdF*, was identified by Tn5 transposon mutagenesis and was sequenced [29]. The product of the cleavage, 4,5,9,10-diseco-3-hydroxy-5-9-17-trioxoandrosta- 1(10),2-diene-4-oic acid, is hydrolysed by TesD in *C. testosteroni* [164], and by HsaD in *M. tuberculosis* [165,166] and *R. jostii* [55], yielding 3aα-H-4α(3′-propanoate)7a-β-methylhexahydro-1,5-indanedione (HIP) and 2-hydroxy-2,4-hexadienoic acid (Figure 7).

In *C. testosteroni*, the catabolism of 2-hydroxy-2,4-hexadienoic acid is carried out by the products of the genes *tesEFG* [167]. Thus, 2-hydroxy-2,4-hexadienoic acid is the substrate of the hydratase TesE leading to the formation of 4-hydroxy-2-oxohexanoate, which then undergoes an aldol cleavage catalysed by TesG aldolase, yielding pyruvate and propionaldehyde. Finally, propionaldehyde is the substrate of an acylating aldehyde dehydrogenase, TesF, producing propionyl-CoA, which enters into the central metabolism (Figure 7). Orthologous genes have been identified in the *M. tuberculosis* H37Rv genome, *hsaEFG*, and it has been proposed that the aldolase and dehydrogenase, HsaF and HsaG, interact as a complex to efficiently perform the catalysis [168].

### 3.5. Degradation of B/C Rings

The degradation of HIP starts with its CoA thioesterification by a specific acyl-CoA synthetase (Figure 7), StdA3 in *P. putida* DOC21 [62], ScdA (ORF18) in *C. testosteroni* [169] and FadD3 in Actinobacteria [119,170]. It has been proposed that in *M. tuberculosis*, HIP-CoA is the substrate of IpdF (ScdG/ORF31 in *C. testosteroni*), which reduces the 5′-keto substituent to a hydroxyl group, leading to the formation of 5-hydroxy-HIP (Figure 7). This compound is then subjected to a β-oxidative process for the elimination of the propionyl-CoA side chain, yielding 3aα-*H*-4α(3′-carboxyl-CoA)-5-hydroxy-7aβ-methylhexahydro-1-indanone (Figure 7). The enzymes involved in this β-oxidation in *M. tuberculosis* are unknown, with the exception of FadE30, which has been proposed to be the acyl-CoA dehydrogenase involved in this process [36,171]. However, in *C. testosteroni*, it has been proposed that this enzymatic reaction is carried out by a heteromeric acyl-CoA dehydrogenase, ScdC1C2 (ORF28,30), and the product of this reaction is the substrate of hydration of the double bond, in a reaction catalysed by an enoyl-CoA hydratase, ScdD (ORF32; Figure 7) [172,173,174]. (7aS)-7a-methyl-1,5-dioxo-2,3,5,6,7,7a-hexahydro-1*H*-indene-carboxyl-CoA, in which the rings C and D remain intact, is then produced by two reactions catalysed by IpdC in *M. tuberculosis* (ScdK/ORF4 in *C. testosteroni*), which introduces a double bond in the C ring, and IpdF in *M. tuberculosis* (ScdG/ORF31 in *C. testosteroni*), which oxidises the 5-OH group (Figure 7) [171,172,173,174]. However, the order of these two reactions has not been determined. Ring D is now opened, previously to ring C, through a hydrolytic reaction mediated by a crotonase, EchA20 (ScdY/ORF5 in *C. testosteroni*) [171,174], producing (*R*)-2-(2-carboxyethyl)-3-methyl-6-oxocyclohex-1-ene- 1-carboxyl-CoA. This molecule is the substrate for the hydrolytic cleavage of ring C, catalysed by IpdAB (ScdL1L2/ORF1,2 in *C. testosteroni*) by a retro-Claisen hydrolysis (Figure 7) [175,176]. The product of the opening of both the C and D rings is the substrate of a thiolase (putatively Fad6), resulting in a molecule of acetyl-CoA and 4-methyl-5-oxo-octanedioyl-CoA (Figure 7) [171,177]. It has been proposed that this last intermediate undergoes a β-oxidation process, starting with a desaturation catalysed by an acyl-CoA dehydrogenase, FadE32, or by the heteromeric Fad31-FadE32 in *Mycobacterium* [171]. In *C. testosteroni*, the enoyl-CoA hydratase involved in the next reaction of this β-oxidation process, ScdN (ORF3), has been identified [177]. As final products of the β-oxidation, a molecule of acetyl-CoA would be released together with 2-methyl-β-ketoadipyl-CoA, which could be cleaved to propionyl-CoA and succinyl-CoA (Figure 7) by a mechanism analogous to the final step in the catabolism of aromatic compounds through the β-ketoadipate pathway [178].

It is important to mention that in the degradation of bile acids in *P. putida* DOC21, the hydroxyl substituents present at C_7_ and/or C_12_ in the different bile acids are maintained during the degradative process. Thus, a *P. putida* DOC21 mutant lacking StdA3, the ATP-depending acyl-CoA synthetase activating HIP, when cultured in the presence of chenodeoxycholic acid accumulates 3′(*R*)-hydroxy-HIP in the culture broth. When deoxycholic acid is used, 7β-hydroxy-HIP accumulation is observed, and in the presence of cholic acid, 3′(*R*),7β-dihydroxy-HIP is accumulated [62]. For *R. jostii* RHA1, it has been proposed that *echA13*, a gene included in the HIP catabolic gene cluster, could codify a function for the removal of the hydroxyl group at position 7β [57]. On the other hand, maintaining the hydroxyl group in the 3′ position in the aliphatic chain of HIP could potentially generate, after CoA thiosterification of the molecule, a C_3_-hydroxy-intermediate from the first β-oxidation round, avoiding the need for an acyl-CoA dehydrogenase, FadE30, and enoyl-CoA hydratase, in the formation of 3aα-*H*-4α(3′-carboxyl-CoA)-5-hydroxy-7aβ-methylhexahydro-1-indanone.

A bioinformatics approach, using selected characteristic genes from this pathway described from model organisms against genomes deposited in public databases, made it possible to identify 265 putative steroid degraders within only the Actinobacteria and Proteobacteria, from different habitats, including eukaryote hosts, soil, and aquatic environments [93]. This study allowed for a comparison of the organisation of the genetic clusters encoding this pathway in different organisms, and with different steroid use profiles (sterols, testosterone and bile acids). This study showed that most of the genes coding the whole 9,10-*seco* pathway are randomly located in different low number large clusters in the bacterial chromosome. However, there are some exceptions, such as *M. tuberculosis* H37Rv, where the predicted catabolic genes are mainly located in a single cluster (80 genes in a region of about 50 kb; Figure 4) [55,93]. *Rhodococcus* spp. have their cholic acid catabolism genes grouped in a separate cluster not close to the cholesterol catabolism genes, and lacking C/D-rings catabolic genes (i.e., *R. jostii* RHA1, Figure 4). Even so, the *R. equii* genome has a single cluster containing the putative cholic acid and cholesterol catabolic genes [93]. In general, the location of these clusters is chromosomal, however, Bergstrand et al. [93] identified some exceptions, where the genes are located on plasmids, such as the putative cholic acid A/B-ring degradation genes located on pRHL1 from *R. jostii*, and a gene cluster encoding putative enzymes involved in testosterone and/or cholic acid catabolism in two strains belonging to the genus *Novosphingobium*. The association of genes coding this pathway opens the possibility of the horizontal transfer of catabolism of steroids between bacteria.

Genes involved in the catabolism of cholesterol through the 9,10-*seco* pathway are part of the core genome characterising most of the genera of the Corynebacterinae. However, the cholic acid pathway, although conserved in *Rhodococcus* spp., is seldom found in closely related genera. This restricted distribution of the actinobacterial bile acid pathway suggests that its origin could be through the duplication of a pre-existing cholesterol pathway in an ancestor of the genus *Rhodococcus*. This could be the reason why genes encoding A/B-ring degradation are found in gene clusters for both cholesterol and bile acid pathways. Surprisingly, genes for C/D-ring degradation are found only in cholesterol pathway clusters, indicating that they have not been duplicated, or have been lost through the evolution of the strains.

Proteobacteria are unable to degrade sterols with alkyl side chains [29,60]. This could be due to the absence of Cyp125 and/or Cyp142 orthologs allowing side chain functionalisation and degradation, as well as the lack of a *mce*-like transport system for sterol uptake. The wide distribution of 9,10-*seco* pathway genes in Actinobacteria contrasts with the scarcity of androgen/bile acids pathway genes in Proteobacteria genomes. However, an exception to this could be the genus *Comamonas*, in which this pathway seems to be part of the genome core [93].

Although the bile acid and the androgen catabolic pathways of Proteobacteria are highly similar to the proposed pathways for actinobacterial cholesterol and cholic acid catabolism, the encoding genes show low sequence similarity. This could be explained by: (i) convergent evolution, where these genes evolved independently in both taxonomic groups [179]; or (ii) divergent evolution, with the genes having an actinobacterial origin, and being disseminated through horizontal gene transfer to the other taxon [93]. Although some interesting articles assess the phylogeny of some critical genes from these pathways [93,141,158], complementary studies will be necessary to clarify this issue.

### 3.6. Regulation of 9,10-seco Pathway

In *M. tuberculosis*, the regulation of cholesterol degradation is carried out by two TetR-like transcriptional repressors, KstR and KstR2 [73,180]. These repressors regulate the expression of particular genes after binding specific intermediates from cholesterol degradation. Thus, KstR unlocks the expression of the genes encoding the membrane transport system of cholesterol, as well as enzymes involved in side chain catabolism and the opening and removal of steroidal A and B rings through binding to 3-hydroxy-cholest-5-en-26-oyl-CoA [181]. This regulatory protein is not only involved in cholesterol catabolism, as the KstR regulon comprises 74 genes in *M. tuberculosis*, some of them involved in growth on palmitate, suggesting that KstR may control metabolism of lipids in this bacterium [73]. KstR may act as a de-repressor by binding to molecules other than 3-hydroxy-cholest-5-en-26-oyl-CoA. Moreover, in *M. smegmatis*, it has been demonstrated that cholesterol and its first catabolic intermediate, cholest-4-en-3-one, were unable to induce the release of KstR from proposed promoter regions in the regulon. Furthermore, in *M. smegmatis*, this regulator remains unaffected by other cholesterol catabolic intermediates, such as AD and ADD [182]. By contrast, KstR2 also downregulates the pool of genes needed for C and D ring degradation, and de-represses their expression by binding HIP-CoA as a ligand [183,184].

The regulation of the 9,10-*seco* pathway mediated steroid catabolism in Proteobacteria has mainly been studied in *C. testosteroni*. A gene called *tesR* in strain TA441, and *teiR* in strain ATCC11996, in the steroid degradation gene clusters, encode a transcriptional regulator needed for induction of 3,17β-HSD dehydrogenase, 3α-hydroxysteroid dehydrogenase, 3-ketosteroid-Δ^1(2)^-dehydrogenase, 3-ketosteroid-Δ^4(5α)^-dehydrogenase and most of the identified steroid degradation genes [185,186,187,188]. It has been reported that TesR/TeiR is a membrane protein with a polar location in cells involved in chemotaxis, and mediates steroid sensing and metabolism via kinase activity, which likely triggers a cascade of phosphorylation events that induce the expression of steroid catabolising enzymes [187].

In addition, two negative regulator genes, *repA* and *repB*, have been identified close to *hsdA*, the gene coding for 3α-hydroxysteroid dehydrogenase. The products of these genes repress *hsdA* expression: (i) at the transcriptional level, by binding to *hsdA* promoter sequences in response to the presence of steroids (RepA) [189]; and (ii) by interfering with its translation, by binding to the mRNA of 3α-hydroxysteroid dehydrogenase (RepB) [190]. Moreover, HsdR, a LysR-type transcriptional repressor, activates transcription of the *hsdA* gene in *C. testosterone*, dependent on decreased repression by RepA [191,192].

By contrast, in *C. testosteroni* ATCC11996, a complex network regulating the expression of the 3,17β-HSD gene, *βhsd*, has been proposed, with several transcriptional repressors, PhaR [193], LuxR [194], TetR [195], and BRP [196] having been described. In knock-out mutants for *phaR*, *luxR*, *tetR*, and *brp*, the basal expression levels of *βhsd* did not increase with reference to the wild type strain; however, on the addition of testosterone there was a several-fold increase in expression levels compared to the wild type, suggesting that these regulators function as repressors of *βhsd* expression [193,194,195,196]. This implies the existence of a complex controlling *βhsd* expression, which is regulated by testosterone. In addition, the expression of 3α-HSD was also increased in the *brp* knock-out mutant, indicating that BRP represses the expression of both *hsdA* and *βhsd* in the presence of testosterone [196].

## 4. The 4,5-*seco* Pathway

Compared with the 9,10-*seco* pathway for sterols, bile acids, and androgen aerobic degradation, current knowledge on estrogen degradation pathways is very limited. The partial characterisation of the aerobic degradation of 17β-estradiol by *Sphingomonas* sp. strain KC8, an obligate aerobic alpha-proteobacterium isolated from wastewater [197], has allowed a new pathway for aerobic estrogen degradation, the 4,5-*seco* pathway, to be proposed [198].

**Figure 8:**
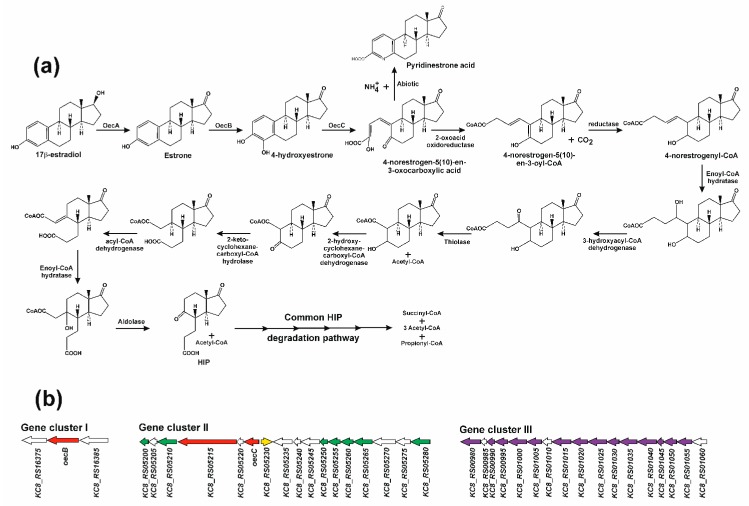
Metabolism of 17β-estradiol in *Sphingomonas* sp. KC8. (**a**) Reactions of the 4,5-*seco* pathway. HIP, 3aα-H-4α(3′-propanoate)7a-β-methylhexahydro-1,5-indanedione. (**b**) Genetic organisation of the three clusters codifying the enzymes required for 17β-estradiol assimilation (Genome Accession NZ_CP016306). Annotation from the genome: KC8_RS16375, putative dioxygenase; KC8_RS16385, Rieske (2Fe 2S) protein; KC8_RS05200, MaoC dehydratase; KC8_RS05205, hypothetical protein; KC8_RS05210, acyl-CoA dehydrogenase; KC8_RS05215, ferredoxin oxidoreductase; KC8_RS05220, VOC family protein; KC8_RS05230, TetR transcriptional regulator; KC8_RS05235, cytochrome P450; KC8_RS05240, hypothetical protein; KC8_RS05245, lipid-transfer protein; KC8_RS05250, MaoC dehydratase; KC8_RS05255 and KC8_RS05260, enoyl-CoA hydratases; KC8_RS05265, acetyl-CoA acetyltransferase; KC8_RS05270, 3-hydroxy-3-mthylglutaryl-CoA synthase; KC8_RS05275, Short-chain oxidoreductase; KC8_RS05280, acyl-CoA dehydrogenase; KC8_RS00980, CoA acyltransferase; KC8_RS00985, steroid Δ-isomerase; KC8_RS00990, MaoC dehydratase; KC8_RS00995, short-chain dehydrogenase/reductase; KC8_RS01000, acyl-CoA dehydrogenase; KC8_RS01005, short-chain oxidoreductase; KC8_RS01010, phenylacetic acid degradation protein PaaY; KC8_RS01015, acetyl-CoA acetyltransferase; KC8_RS01020, acyl-CoA dehydrogenase; KC8_RS01025, acyl-CoA dehydrogenase; KC8_RS01030, enoyl-CoA hydratase; KC8_RS01035, monooxygenase; KC8_RS01040, lipid-transfer protein (Ltp); KC8_RS01045, thiolase; KC8_RS01050, CoA transferase, β-subunit; KC8_RS01055, CoA transferase, α-subunit; KC8_RS01060, *meta*-dioxygenase. Genes encoding key enzymes for A ring degradation are depicted in red; genes coding β-oxidation related enzymes putatively catalyzing degradation of A/B ringare indicated in green; genes for C/D-ring degradation are depicted in purple.

*Sphingomonas* KC8 codes for a gene for 3β,17β-hydroxysteroid dehydrogenase (*oecA*) that is responsible for the transformation of 17-hydroxy from 17β-estradiol and testosterone to their respective oxo-derivatives. This gene was similarly expressed when this strain was cultured on 17β-estradiol and testosterone, although testosterone is degraded by this strain through the 9,10-*seco* pathway [199]. OecA converts 17β-estradiol into estrone (Figure 8). However, this gene does not cluster with other steroid-degrading genes in the genome of *Sphingomonas* KC8. An ortholog of this gene has been characterised in *P. putida* SJTE-1, a strain isolated from sludge, which can use 17β-estradiol as a sole carbon source [200,201].

Additionally, other genetic clusters involved in estrogen catabolism have been identified in the genome of *Sphingomonas* KC8 (Figure 8). In gene cluster I, an *oecB* gene encodes a flavin-dependent estrone 4-hydroxylase, which introduces a hydroxyl group at C_4_ in the aromatic ring, yielding 4-hydroxyestrone. In gene cluster II, *oecC* codes for a 4-hydroxyestrone 4,5-dioxygenase that catalyses the *meta*-cleavage of the aromatic ring, creating 4-norestrogen-5(10)-en-3-oxo-carboxylic acid. This meta-cleavage product is unstable and undergoes an abiotic recyclisation, producing pyridinestrone acid, a dead-end byproduct that accumulates in the culture broth of *Sphingomonas* KC8 when grown on 17β-estradiol (Figure 8) [198]. However, the metabolic pathway continues by using a 2-oxoacid oxidoreductase that catalyses the decarboxylation of this meta-cleavage product, as well as the thioesterification of the resulting molecule with CoA, to produce 4-norestrogen-5(10)-en-3-oyl-CoA (Figure 8). The gene encoding this 2-oxoacid oxidoreductase is also found in gene cluster II. This cluster also includes genes encoding β-oxidation-like enzymes. Metabolic intermediates in estrone degradation have allowed the identification of products of these genes involved in the transformation of the cleavage product from 4-hydroxyestrone to HIP (Figure 8). In addition, the existence of a gene encoding for a 3-hydroxy-3-methylglutaryl-CoA synthase-like protein in this cluster suggests its involvement in estrogen catabolism [202].

In summary, *oecA* and genes from clusters I and II are likely to be involved in estrogen A/B-ring degradation to HIP in *Sphingomonas* KC8. In the genome of this strain, a third cluster (cluster III) related to steroid degradation has also been identified (Figure 8). It contains genes similar to those proposed in *C. testosteroni* for C/D-ring degradation. Thus, orthologs of *echA20*, *ipdB*, and *ipdA* from *M. tuberculosis* have been found (Figure 8), and they are expressed during the aerobic growth of *Sphingomonas* KC8 on testosterone and 17β-estradiol, being involved in the transformation of HIP into general catabolites [198,202]. Similar clusters have been identified in other estrogen-degrading aerobes, such as *Altererythrobacter estronivorus* MH-B5 [203] and *Novosphingobium tardaugens* NBRC 16725 [204].

**Figure 9:**
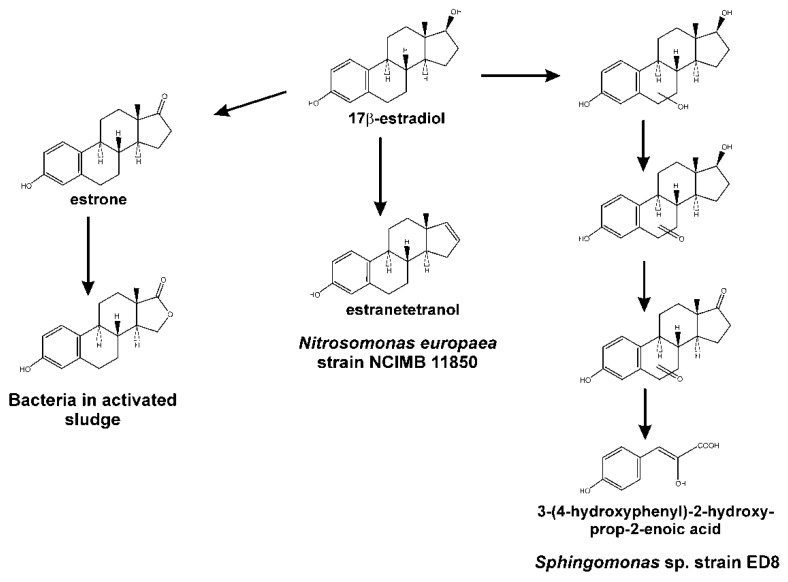
Metabolic alternatives to the 4,5-*seco* pathway proposed for estrogen mineralisation/biotransformation in different bacterial species.

It has been proposed that the 4,5-*seco* pathway is highly prevalent, since: (i) the dead-end product pyridinestrone is found in wastewater treatment plants exposed to estrogens, and (ii) the gene *oecC* has been identified in bacteria isolated from these environments [198,205]. Alternative estrogen degradation pathways and metabolites have been proposed (Figure 9), although there is no biochemical or genetic evidence identifying their significance. Thus, in *Sphingomonas* sp. strain ED8, a pathway has been proposed, involving hydroxylation at different positions of the saturated ring of 17β-estradiol. This is supported by the detection of hydroxyl-17β-estradiol, keto-17β-estradiol, keto-estrone and 3-(4-hydroxyphenyl)-2-hydroxy-prop-2-enoate in the culture broth of this bacterium when 7β-estradiol was used as the sole carbon source (Figure 9). The appearance of this last compound suggested that catabolism of 17β-estradiol through this pathway takes place by opening the rings B, C, or D [206]. Another metabolic alternative for 17β-estradiol mineralisation has been proposed in *Nitrosomonas europaea*, based on the appearance of estra-1,3,5(10),16-tetraen-3-ol (estratetraenol) after dehydratation of ring D at C17 position (Figure 9). It was suggested that this strain could further metabolise this intermediate to non-estrogenic compounds [207]. The finding, in activated sludge, of a new intermediate from 17β-estradiol derived from estrone and containing a lactone in ring D, opens the possibility of a new estrogen catabolic pathway used for the mineralisation of this kind of steroid (Figure 9) [208].

## 5. The Anaerobic 2,3-*seco* Pathway

**Figure 10:**
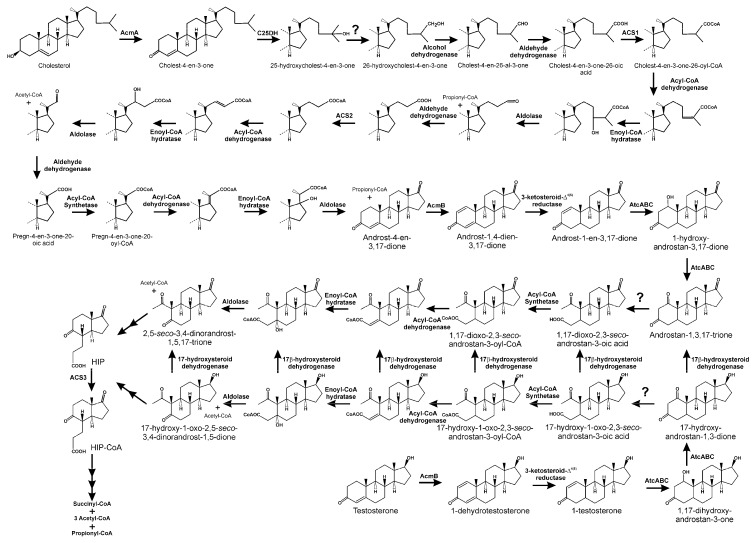
Anaerobic catabolism of cholesterol, in *Sterolibacterium denitrificans*, and testosterone, in *Steroidobacter denitrificans*, by the 2,3-*seco* pathway. Putative points of convergence between both metabolic mechanisms are also suggested.

Several denitrifying Proteobacteria that degrade steroids, cholesterol, and testosterone under anaerobic conditions have been characterised [63,65,209]. Among them, *Sterolibacterium denitrificans* (*Stl*. *denitrificans*) and *Steroidobacter denitrificans* (*Std. denitrificans*) have been used as model organisms for studying anaerobic steroid metabolism [63,65]. The pathway involved in anoxic steroid catabolism, although having some similarities with the 9,10-*seco* pathway, uses dioxygen-independent reactions to degrade the steroidal core. It is referred to as the 2,3-*seco* pathway [210,211,212]. Similar to the aerobic metabolism performed through the 9,10-*seco* pathway, the anaerobic degradation of cholesterol by *Stl. denitrificans* is initiated by the oxidation of ring A using AcmA, a dehydrogenase/isomerase belonging to the short-chain dehydrogenase/reductase superfamily, yielding 4-cholesten-3-one (Figure 10) [64,213].

In a second step, the anaerobic degradation of cholesterol C_17_ side chain starts with an oxygen-independent water-dependent hydroxylation at the tertiary C_25_ atom of the side chain of C_27_ esteroid substrates, resulting in the formation of a tertiary alcohol, 25-hydroxy-4-cholesten-3-one (Figure 10) [213]. This hydroxylation is carried out by a molybdopterin-containing enzyme, C25DH, belonging to the dimethyl sulfoxide dehydrogenase molybdoenzyme family. This enzyme consists of three different subunits, an α-subunit containing the molybdo-*bis*(pyranopterin guanine dinucleotide) cofactor in the active site and an iron–sulfur cluster [4Fe-4S], associated with electron-transfer machinery composed of β-subunits that contain three [4Fe-4S] and one [3Fe-4S] iron–sulfur clusters, and ϒ-subunits associated with one heme b. Notably, the genome of *Stl. denitrificans* Chol1-S contains seven paralogs that code for putative α-subunits of S25DH-like dehydrogenases, although there are few genes putatively encoding the β- and ϒ-subunits, indicating that molybdoenzymes share common βϒ-components, but have different α-subunits [214,215]. *Stl. denitrificans* is able to not only degrade cholesterol, but also β-sitosterol, stigmasterol, and ergosterol. Surprisingly, S25DH used in cholesterol catabolism is unable to hydroxylate any of the 4-en-3-one analogues of these sterols [214,216]. However, several S25DHs carrying different α-subunits have been functionally characterised, and are able to activate those sterols [217].

**Figure 11:**
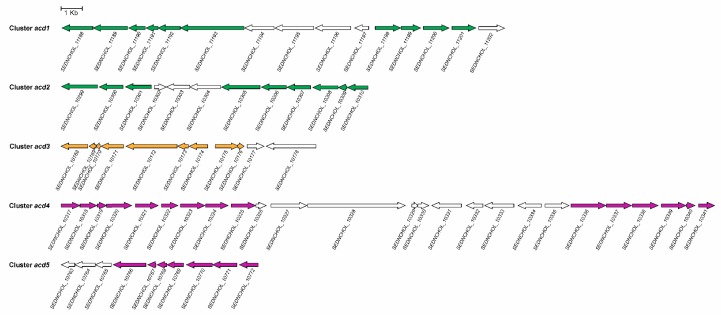
Genetic organisation of the genes encoding 2,3-*seco* pathway functions for cholesterol degradation in *Sterolibacterium denitrificans*. Annotation in the genome (accession LT837803): SEDNCHOL_11188, aldehyde dehydrogenase; SEDNCHOL_11189, acyl-CoA synthetase 1 (ACS1); SEDNCHOL_11190, short-chain dehydrogenase; SEDNCHOL_11191, SEDNCHOL_11192, and SEDNCHOL_11193 steroid C25 dehydrogenase, γ-, β- and α- subunit, respectively; SEDNCHOL_11194 and SEDNCHOL_11195, proteins with unknown functions; SEDNCHOL_11196, AcmB; SEDNCHOL_11197, putative transcriptional regulatory protein; SEDNCHOL_11198, putative aldolase; SEDNCHOL_11199, enoyl-CoA hydratase; SEDNCHOL_11200 and SEDNCHOL_11201, acyl-CoA dehydrogenases; SEDNCHOL_11202, oxidoreductase; SEDNCHOL_10299, acyl-CoA synthetase 2 (ACS2); SEDNCHOL_10300 and SEDNCHOL_10301, acyl-CoA dehydrogenases; SEDNCHOL_10302, protein of unknown function; SEDNCHOL_10303, putative metallo-β-lactamase; SEDNCHOL_10304, phytoene dehydrogenase-like protein; SEDNCHOL_10305, putative C_22_ acyl-CoA synthetase; SEDNCHOL_10306 and SEDNCHOL_10307, acyl-CoA dehydrogenases; SEDNCHOL_10308, aldolase; SEDNCHOL_10309 and SEDNCHOL_10310, enoyl-CoA hydratases; SEDNCHOL_10168, CoA transferase; SEDNCHOL_10169, plasmid stabilization system; SEDNCHOL_10170 and SEDNCHOL_10171, proteins of unknown function; SEDNCHOL_10172, SEDNCHOL_10173, and SEDNCHOL_10174, A, B, and C subunits of AtcABC, respectively; SEDNCHOL_10175 and SEDNCHOL_10176, proteins of unknown function; SEDNCHOL_10177; putative electron-transfer flavoprotein, β-subunit; SEDNCHOL_10178, protein of unknown function; SEDNCHOL_10317, SEDNCHOL_10318, and SEDNCHOL_10321, IpdABC-like proteins; SEDNCHOL_10319, probably subunit of benzoylsuccinyl-CoA thiolase; SEDNCHOL_10320, Propanoyl-CoA C-acyltransferase; SEDNCHOL_10322, enoyl-CoA hydratase; SEDNCHOL_10323 and SEDNCHOL_10324, acyl-CoA dehydrogenases; SEDNCHOL_10325, thiolase; SEDNCHOL_10326, MarR family transcriptional regulator; SEDNCHOL_10327, outer membrane protein; SEDNCHOL_10328, putative Filamentous hemagglutinin family N-terminal domain containing protein; SEDNCHOL_10329, protein of unknown function; SEDNCHOL_10330, N-acetyltransferase; SEDNCHOL_10331, RseB; SEDNCHOL_10332, enoyl-CoA hydratase; SEDNCHOL_10333 and SEDNCHOL_10334, proteins of unknown function; SEDNCHOL_10335, CoA transferase; SEDNCHOL_10336, putative IpdC; SEDNCHOL_10337 and SEDNCHOL_10338, CoA transferases; SEDNCHOL_10339, acetyl-CoA acetyltransferase; SEDNCHOL_10340, protein of unknown function; SEDNCHOL_10341, enoyl-CoA hydratase; SEDNCHOL_10763, putative 2-phospho-L-lactate guanylyltransferase; SEDNCHOL_10764, 2-phospho-L-lactate transferase; SEDNCHOL_10765, Coenzyme F420:L-glutamate ligase; SEDNCHOL_10766, acyl-CoA synthetase 3 (ACS3); SEDNCHOL_10767, steroid Δ–isomerase; SEDNCHOL_10768, enoyl-CoA hydratase; SEDNCHOL_10769, short chain dehydrogenase; SEDNCHOL_10770 and SEDNCHOL_10771, acyl-CoA dehydrogenases; SEDNCHOL_10772, short chain alcohol dehydrogenase. Genes encoding enzymes involved in cholesterol side chain degradation are shown in green; genes participating in A ring degradation are shown in orange; and in purple is shown genes coding for ring C/D degradation.

The next step in the anoxic degradation of cholesterol involves an unprecedented isomerisation of the hydroxyl group from the tertiary C_25_ to the primary C_26_, catalysed by a yet unknown enzyme (Figure 10) [86,218]. Further degradation proceeds via oxidation of C_26_ primary alcohol to a carboxylate by the action of a putative cholesterol-induced alcohol dehydrogenase and aldehyde dehydrogenase (Figure 10) [216]. This carboxylic derivative is activated to a C_26_-oyl-CoA thioesterifed molecule by a specific ATP-depending acyl-CoA synthetase, followed by a modified β-oxidation-like reaction sequence, with some steps similar to those of bile acid catabolism through the 9,10-*seco* pathway, yielding AD, two propionyl-CoAs, and one acetyl-CoA (Figure 10) [216,219]. Thus, in the genome of *Stl. denitrificans*, two gene clusters, *acd1* and *acd2*, contain the genes that, after induction by cholesterol, code for functions needed for cholesterol side chain elimination (Figure 11). Amazingly, although more of the intermediates of side chain degradation have been identified, no 3-oxoacyl-CoA intermediates have been found, which correlates with the lacking of genes specifically involved in the thiolytic β-oxidative processes (3-hydroxyacyl-CoA dehydrogenases and thiolases). Alternatively, the two aldolases and an aldehyde dehydrogenase in the *acd1* and *acd2* clusters indicate that the release of propionyl-CoA and acetyl-CoA molecules from degradation of the side chain occurs via aldolytic cleavage [216,219].

In vitro assays using cell-free extracts indicate the existence of an FAD-containing 4-cholesten-3-one-Δ^1(2)^-dehydrogenase (AcmB) in *Stl. denitrificans*, catalysing Δ^1(2)^-desaturation of 4-cholesten-3-one to 1,4-cholestadien-3-one (Figure 10) [220]. However, the topological location of AcmB on the cytoplasmic side of the inner membrane and 4-cholesten-3-one in the cellular periplasm puts this proposed reaction in doubt. Thus, AcmB may be a 3-ketosteroid-Δ^1(2)^-dehydrogenase, with AD, obtained after complete removal of the lateral side chain from 4-cholesten-3-one, being its physiological substrate, leading to the formation of ADD. This later compound is then specifically reduced to 1-androsten-3,17-dione, which acts as a substrate for AtcABC, a bifunctional molybdopterin-containing hydratase/dehydrogenase, which introduces a water molecule to the double bond between C_1_ and C_2_, leading to the formation of a hydroxyl group at C_1_. Later, this hydroxyl group is oxidised to an oxo-group, releasing androstan-1,3,17-trione (Figure 10) [212]. The A ring of this compound is then cleaved, resulting in 1,17-dioxo-2,3-*seco*-androstan-3-oic acid, which would be activated by a yet unknown acyl-CoA synthetase (Figure 10) [86,212,218]. It has been suggested that acetyl-CoA could be released from 1,17-dioxo-2,3-*seco*-androstan-3-oyl-CoA through an aldolytic cleavage, to produce 2,5-*seco*-3,4-dinorandrost-1,5,17-trione [213]. Although the mechanism for cleavage of the B-ring remains unknown, HIP, the predicted product generated from 2,5-*seco*-3,4-dinorandrost-1,5,17-trione, has been identified (Figure 10) [216].

In *Stl. denitrificans*, genes involved in ring A cleavage have been found in the cluster *acd3* (Figure 11), although no candidates encoding a B-ring cleaving hydrolase or its degradation to HIP have been identified. Clusters *acd4* and *acd5* contain all the genes required for the integration of HIP into the central metabolism (Figure 11) [216].

The anaerobic degradation of the androgen testosterone has mainly been studied in model microorganism *Std. denitrificans* DSMZ 18526 [210,211,221,222,223]. The pathway used by this denitrifying strain generates analogous intermediates to those of the 2,3-*seco* pathway involved in cholesterol catabolism in *Stl. denitrificans*, and common steps in these two pathways are catalysed by orthologous enzymes (Figure 10) [212].

Thus, testosterone is initially transformed into 1-dehydrotestosterone by a 3-ketosteroid Δ^1(2)^-dehydrogenase/reductase (Figure 10). This compound is then transformed to 1-testosterone in a process catalysed by a 3-ketosteroid Δ^4^-dehydrogenase/reductase (Figure 10). AtcABC, the analogous bifunctional molybdoenzyme, in this case a 1-testosterone hydratase/dehydrogenase, catalyses the C1-C2 hydration reaction and the subsequent oxidation, leading to the formation of 17-hydroxy-androstan-1,3-one (Figure 10) [211,212]. It has been proposed that the process continues through the hydrolytic cleavage of this last compound, giving rise to 17-hydroxy-1-oxo-2,3-*seco*-androstan-3-oic acid (Figure 10). However, the hydrolase that would catalyse this process remains unidentified. Taking into account that 17-hydroxy-2,5-*seco*-3,4-dinorandrost-1,5-dione has been identified in cultures of *Std. denitrificans* growing in media containing testosterone as a carbon source, the metabolic steps for the transformation of 17-hydroxy-1-oxo-2,3-*seco*-androstan-3-oic acid into this compound have been proposed. Thus, the acid is first activated to a coenzyme A thioester, and after the introduction of a double bond and its hydration, a retroaldolic reaction, a molecule of acetyl-CoA is released, producing 17-hydroxy-2,5-*seco*-3,4-dinorandrost-1,5-dione (Figure 10). Notably, the use of acyl-CoA dehydrogenase inhibitors impairs the biotransformation of 17-hydroxy-1-oxo-2,3-*seco*-androstan-3-oic acid to 17-hydroxy-2,5-*seco*-3,4-dinorandrost-1,5-dione, reinforcing the participation of a β-oxidation-like mechanism in the processing of the A-ring cleavage product [211]. It has been proposed that, as occurs in cholesterol degradation, this compound will be converted to HIP.

It has to be noted that proposed intermediates in testosterone anaerobic catabolism conserve a hydroxyl group at C_17_ until their convergence at the HIP level. However, this only reflects the fact that these compounds are more prominent in the culture broth than their 17-keto structures, which appear at lower levels. This has resulted in a proposal that a 17β-hydroxysteroid dehydrogenase, or a group of enzymes with this activity, could interconvert the different 17β-hydroxyl compounds and their 17-keto intermediates [211,223]. Undoubtedly, if the catabolism of steroids through this pathway occurs by the formation of HIP at some stage, the keto present in the D-ring should be evident.

Taking into account that the aerobic and anaerobic degradation of steroids converge at HIP, it might be expected that the same metabolic mechanisms are used for its subsequent degradation. This hypothesis is reinforced by the conservation of genes in the genomes of microbial steroid degraders [216,219].

## 6. Biotechnological Interest in Steroid-Degrading Microorganisms

In addition to the relevance that steroid-degrading microorganisms have for the environment and maintaining the carbon cycle, their potential use in the pharmaceutical industry has to be highlighted. Classical pharmaceutical production processes for steroids have been carried out by extraction from plant or animal sources, by full organic synthesis, or by a combination of chemical and enzymatic synthesis. With exceptions, extraction from animals or plants is an inefficient large-scale production method. Full chemical and chemical-enzymatic syntheses are often multistage and very expensive in time, labor, and energy, as well as posing a potential risk for the environment.

As an alternative, the bioconversion of steroids from low-cost precursors (i.e., phytosterols) with genetically tailored microorganisms is becoming the method of choice for industrial production. The development of these biotechnological approaches, which are more cost-effective and environmentally friendly, have been stimulated due to the growing demand for steroid pharmaceuticals. This approach is classically developed with heterologous gene expressing transgenic bacteria, yeast, and fungi, or with specific strains empirically showing a specific reaction. Steroid degrading strains, or specific mutants tailored from them, has given rise to a new source of syntons (mainly AD, ADD, or 9-hydroxy-derivatives) or genes/enzymes useful for steroid transformation.

As far as we know, today there have been no studies focused in the development of biodegradative strategies for environmental steroid amendment. Despite the great interest that the elimination of steroids can have due to the risk that the release of these compounds to the environment has for flora and wild animal reproduction, and the potential effect on human beings, the biotechnological approaches to these applications have not been developed yet.

Although our knowledge about the metabolic mechanisms for catabolism of sterols, bile acids, testosterone, or 17β-estradiol has increased in recent years, some black holes need to be clarified. The transport of steroids across cell walls and membranes in some of the bacterial groups able to metabolize steroids, rate-limiting steps in the pathways, or global regulation and integration of these pathways at intermediate metabolism should be more deeply elucidated. Moreover, there are many steroid compounds released to the environment for which it is not yet known how they could be integrated into carbon cycles.

Study of metagenomics communities will allow the identification of novel steroid biodegraders. The isolation and characterization of individual microorganisms with the ability for steroid degradation will increase the pool of genes, enzymes, and metabolic strategies for environmental steroid amendment. It is to be expected that this set of knowledge, together with technologies developed in other disciplines, will allow the design of new bacteria, or communities of them, suitable for use in the biodegradation of these molecules. Thus, for instance, system biology will allow the design of interacting communities of microorganisms for the biodegradation of different steroids or the integration of new metabolic circuits inside a bacteria; metabolomics will permit the improvement of metabolic fluxes, ensuring a high efficiency in the catabolism of steroids; and synthetic biology would allow the design and expression of improved or new activities based on mammalian, fungi, or gut microbiota steroid metabolism. In sum, the open future for a biodegradative approach to remove environmentally released steroids is expected to be a promising reality.

## Figures and Tables

**Table 1:** Model strains widely used in the studies that have allowed the characterization of the metabolic mechanisms used for steroid degradation. Steroids used in these studies are also indicated.

	Strain		Steroids Used	Reference Genome
9,10-*seco* pathway	*Mycobacterium*	*tuberculosis* H37Rv	Cholesterol	NC_000962
		*smegmatis* mc^2^ 155	Cholesterol	NC_008596
	*Rhodococcus*	*neoaurum* ATCC 25795	Cholesterol	NZ_JMDW00000000
			β-sitosterol	
			Stigmasterol	
			Campesterol	
		*equi* 103S	Cholesterol	NC_014659
		*rhodochrous* DSM43269	Cholesterol	unpublished
			β-sitosterol	
			Campesterol	
		*jostii* RHA1	Cholesterol	NC_008268
			Cholic acid	
		*erythropolis* SQ1	Cholesterol	unpublished
	*Gordonia*	*neofelifaecis* NRRL B-59395	Cholesterol	NZ_AEUD00000000
		*cholesterolivorans* Chol-3	Cholesterol	unpublished
			Ergosterol	
			Stigmasterol	
	*Comamonas*	*testosteroni* TA441	Testosterone	NZ_CP006704
			Cholic acid	
		*thiooxidans* CNB-1	Testosterone	NC_013446
	*Pseudomonas*	*stutzeri* Chol-1	Cholic acid	NZ_AMSL00000000
		*putida* DOC21	Bile acids	unpublished
			Testosterone	
4,5-*seco* pathway	*Sphingomonas*	sp. KC8	17β-estradiol	NZ_AFMP01000000
2,3-*seco* pathway	*Sterolibacterium*	*denitrificans* Chol1S	Cholesterol	LT837803
	*Steroidobacter*	*denitrificans* DSMZ18526	Testosterone	NZ_CP011971
